# Astaxanthin activates the Nrf2/HO-1 pathway to attenuate indoxyl sulfate-induced oxidative stress and DNA damage in renal tubular epithelial cells

**DOI:** 10.3389/fphar.2025.1715462

**Published:** 2026-01-05

**Authors:** Xiaoli Qian, Zhixia Wang

**Affiliations:** 1 Department of Nephrology, Wujin Hospital Affiliated with Jiangsu University, Changzhou, Jiangsu, China; 2 Department of Nephrology, The Wujin Clinical College of Xuzhou Medical University, Changzhou, Jiangsu, China

**Keywords:** chronic kidney disease, astaxanthin, Nrf2/HO-1 pathway, oxidative stress, network pharmacology

## Abstract

Astaxanthin (AST) was investigated for its therapeutic role in chronic kidney disease (CKD) and its underlying mechanisms. Network pharmacology analysis identified 29 overlapping targets of AST and CKD, which were enriched in oxidative stress-related pathways, particularly the Nrf2/HO-1 axis. Molecular docking further confirmed stable binding of AST to hub proteins such as HMOX1, SOD2, and NOS2. *In vitro*, indoxyl sulfate (IS)-treated HK-2 cells were used to establish a CKD injury model. Cell viability was assessed by MTT assay, apoptosis by TUNEL staining and Western blotting, oxidative stress by ROS detection and measurement of glutathione (GSH), superoxide dismutase (SOD), and malondialdehyde (MDA). DNA damage was evaluated by alkaline comet assay and γH2AX expression, while cellular senescence was examined by SA-β-galactosidase staining and p53/p21 expression. IS exposure significantly increased apoptosis, oxidative stress, DNA damage, and senescence, while reducing cell viability, antioxidant capacity, and nuclear Nrf2 expression. AST treatment effectively reversed these changes, improving cell viability and antioxidant defenses, and alleviating apoptosis, ROS accumulation, DNA damage, and senescence. These integrated computational and experimental findings indicate that AST exerts renoprotective effects through coordinated modulation of multiple oxidative stress–related pathways, primarily via activation of the Nrf2/HO-1 signaling axis.

## Introduction

Chronic kidney disease (CKD) is characterized by chronic structural damage and dysfunction of the kidneys caused by a variety of etiological factors, with a duration of more than 3 months (Chen et al., 2019). CKD is one of the most common diseases in the world, and epidemiological surveys have shown that more than 850 million people globally suffer from CKD (Jager et al., 2019). CKD not only has a high prevalence and incidence but also poses a significant threat to human life and safety. In 2016, CKD ranked as the 13th leading cause of death globally, resulting in approximately 2.6 million deaths (Collaborators, 2024). Alarmingly, CKD is expected to rise to the 5th leading cause of death globally by 2040 (Foreman et al., 2018). These statistics indicate the urgency of finding effective treatments for CKD.

Although the pathogenesis of CKD is complex and diverse and multiple pathological processes interact during disease progression, the prevailing view suggests that the accumulation of gut-derived uremic toxins (GUTs) *in vivo* may be a central factor in the occurrence and progression of CKD (Lee et al., 2024). Specifically, GUTs, represented by indoxyl sulfate (IS), hippuric acid, and methylguanidine, are a large group of harmful metabolites produced by intestinal microorganisms and absorbed into the bloodstream through the intestinal wall. In individuals with normal renal function, GUTs are rapidly excreted by the kidneys, thus rarely causing adverse health effects. However, in patients with CKD, the metabolites cannot be efficiently excreted due to impaired renal function, resulting in a gradual accumulation of GUTs in the body. When excessive localized GUTs accumulate in the kidneys, GUTs damage the kidneys in various ways including mediating oxidative stress, activating inflammatory responses, inhibiting anti-aging gene expression, and promoting fibrosis. Such processes can further exacerbate CKD deterioration (Ikee et al., 2020). This bidirectional association between renal dysfunction and intestinal micro-ecological imbalance is considered to be one of the key pathogenic mechanisms of CKD.

Given the impact of GUTs on CKD, in recent years, researchers have attempted to reduce the production of GUTs by killing GUT-producing bacteria, reducing substrate uptake, and inhibiting the function of related enzymes to slow the progression of CKD (Pan and Kang, 2018). However, these methods have limitations, leading to poor therapeutic effects (Evans et al., 2022). For example, due to the complexity of the intestinal microbiota, GUTs can be produced by various bacteria. Therefore, it is difficult to reduce the production of GUTs by killing bacteria. Besides, the precursors of GUTs (mainly amino acids) are widely found in the regular diet, making it challenging to avoid their intake completely. Additionally, multiple physiological functions were performed by GUT-related metabolic enzymes in the body, and the normal operation of other organs can be affected by inhibiting their activities (Lau et al., 2018; Faerber et al., 2023). These issues indicate that protecting the kidneys from GUTs may be an effective way for future CKD treatment, given the difficulty in substantially reducing the production and accumulation of GUTs.

Astaxanthin (AST) is a small-molecule fat-soluble pigment belonging to the carotenoid family, which is widely found in shrimp, crabs, salmon, oysters, algae and fungi (Nishida et al., 2023). AST exhibits a variety of pharmacological effects such as antioxidant, immune enhancement, anti-fibrosis and inflammation regulation (Si and Zhu, 2022), providing protective effects on organs including the central nervous system, liver and lungs (Shen et al., 2014; Zhang et al., 2015; Cunha et al., 2023). The kidneys, as vital organs, can also benefit from AST treatment. Moreover, AST has been demonstrated to effectively delay the progression of diabetic nephropathy and reduce renal ischemia-reperfusion injury (Naito et al., 2021; Qiu et al., 2015). However, there are few studies on the effect of AST on CKD.

To address the unresolved mechanisms of AST in CKD, this study combined network pharmacology prediction with experimental validation. Potential targets and pathways of AST were first identified through bioinformatics analyses and molecular docking. An *in vitro* CKD model was then established by treating HK-2 cells with IS to verify the predicted mechanisms. This integrated approach aimed to elucidate the renoprotective effects of AST and provide new insights into its therapeutic potential in CKD.

## Materials and methods

### Acquisition of AST structure

The chemical structure of AST was retrieved from the PubChem database (https://pubchem.ncbi.nlm.nih.gov/). The compound information, including the CID number and 3D structural file in SDF format, was downloaded. The SDF file was subsequently converted into MOL2 format using the Open Babel software for subsequent molecular docking analysis.

### Prediction of potential targets of AST

The potential targets of AST were predicted using multiple publicly available platforms, including SwissTargetPrediction (http://www.swisstargetprediction.ch/), PharmMapper (http://www.lilab-ecust.cn/pharmmapper/), and Similarity Ensemble Approach (SEA, https://sea.bkslab.org/). To maximize the target coverage while maintaining the specific reliability of the database, we set thresholds based on the scoring systems recommended by each platform: using the targets with a probability value >0 in the SwissTargetPrediction database, as well as the highly-matched targets ranked high in the PharmMapper and SEA databases. After integrating the results from each platform and removing duplicates, we finally obtained a non-redundant list of potential AST targets. Since the similarity algorithms used by these databases are different and cannot be directly compared, using a unified numerical threshold would reduce the prediction sensitivity. Subsequently, through cross-database gene intersection analysis, protein interaction network analysis, and molecular docking verification, further reduction of potential noise was achieved.

### Collection of CKD/Uremic toxin-associated targets

The genes related to chronic kidney disease and uremic toxins were collected from three independent sources. Firstly, genes related to “chronic kidney disease” or “uremia” were retrieved from the GeneCards database (with a correlation score >0) (https://www.genecards.org/) and the OMIM database (http://www.omim.org/). Secondly, differentially expressed genes (DEGs) were identified from the GSE37171 dataset (74 uremic patients and 41 healthy samples) using the limma package, and were screened based on the criterion of |log_2_FC| > 1 and the adjusted P value <0.05. To ensure consistency, all gene names from these three sources were standardized to the official gene symbols approved by HGNC. Then, the gene lists from GeneCards, OMIM, and GEO datasets were integrated, and all duplicate items in different datasets were deleted based on the official gene symbols to generate a completely non-redundant set of genes related to chronic kidney disease/uremia, providing basic data for subsequent analysis.

### Identification of candidate targets and construction of the PPI network

The overlapping genes between AST-related targets and CKD/uremia-associated genes were considered candidate targets. These genes were imported into the STRING database (https://string-db.org/) to analyze protein–protein interactions (PPI), with the species set to “*Homo sapiens*” and a minimum required interaction score >0.4. The interaction data were visualized using Cytoscape software (version 3.9.1). The CytoHubba plugin was employed to identify hub genes according to the degree centrality method, and the Top 10 ranked genes were considered key targets for further analysis.

### GO and KEGG enrichment analysis

Gene Ontology (GO) functional annotation and Kyoto Encyclopedia of Genes and Genomes (KEGG) pathway enrichment analyses were performed for the candidate targets using the R package “clusterProfiler” (version 4.0.5). The threshold for significance was set at an adjusted *P* < 0.05. Enrichment results were visualized using bubble plots and Sankey diagrams to highlight key biological processes and signaling pathways, particularly those related to oxidative stress and the Nrf2/HO-1 signaling axis.

### Molecular docking

Molecular docking was conducted to evaluate the potential interactions between AST and the hub proteins identified in the PPI network. The crystal structures of ALB (1AO6), ANXA5 (1ANW), HMOX1 (1N45), HSP90AA1 (1BYQ), HSP90AB1 (1QZ2), NOS2 (1NSI), and SOD2 (1AP5) were retrieved from the RCSB Protein Data Bank based on the following criteria: (1) crystallographic resolution <2.6 Å to ensure structural accuracy; (2) availability of a defined binding pocket or co-crystallized ligand to represent a biologically relevant conformation; (3) preference for holo or active conformations when annotated; and (4) exclusion of truncated or low-quality entries. Before docking, each protein structure was processed using AutoDock Tools (ADT, version 1.5.7), during which crystallographic water molecules were removed, essential cofactors were retained, polar hydrogens were added, and Kollman charges were assigned. Protonation states of titratable residues were set automatically according to physiological pH, and catalytically important residues—particularly in NOS2—were preserved in their functional ionization states. All receptors were treated as rigid, consistent with standard AutoDock Vina protocol.

The 3D structure of AST was downloaded from PubChem and converted from SDF to MOL2 and then to PDBQT format using Open Babel and ADT. Gasteiger charges were added, and all rotatable bonds were kept active to allow full ligand flexibility during docking. The docking grid for each receptor was defined according to the location of the co-crystallized ligand within the selected PDB entry. For proteins lacking a bound ligand, the grid box was centered on the functionally annotated active site described in the PDB database and supported by published biochemical studies. Grid dimensions were adjusted to fully encompass the binding pocket and allow unrestricted ligand sampling. Docking simulations were performed using AutoDock Vina (version 1.1.2) with default parameters and an exhaustiveness value of 8. The lowest-energy binding mode was selected for each target, and protein–ligand interactions were visualized and analyzed using PyMOL.

### Cell culture, solution preparation and grouping

Human renal cortical proximal tubular epithelial cells (HK-2 cells) were purchased from the American Type Culture Collection (Manassas, VA, United States). Referring to previous studies (Kurosaki et al., 2022; Jeon et al., 2015), HK-2 cells in the logarithmic growth phase were cultured at a density of 1 × 10^5^ cells/mL in Dulbecco’s Modified Eagle’s Medium/Nutrient Mixture F-12 Media (Sunncell, China) containing 10% fetal bovine serum (Beyotime, China) and 1% penicillin/streptomycin solution (Beyotime, China). The cells were pre-incubated for 24 h under serum-containing conditions at 37 °C, and 5% CO_2_. After pre-incubation, the cells were treated with AST solution and/or IS solution under serum-free conditions.

Powdered AST was purchased from MedChemExpress (United States), and powdered IS potassium salt was obtained from Hubei Shishun Biotechnology Co., Ltd. (China). Powdered indoxyl sulfate potassium salt (IS potassium salt) was obtained from Hubei Shishun Biotechnology Co., Ltd. (China), with the chemical name 3-indoxyl sulfate potassium salt (CAS No. 2642-37-7). According to the instructions, the above powders were dissolved in 0.5% dimethyl sulfoxide solution to prepare stock solutions. Then, the solutions were diluted with phosphate buffered saline (PBS) to form different concentrations of AST working solution and IS working solution for experiments.

HK-2 cells were treated with different concentrations of AST working solution (0 μM, 5 μM, 10 μM, 15 μM, 20 μM, and 25 μM) and IS working solution (0 μM, 50 μM, 100 μM, 150 μM, 200 μM, and 250 μM), respectively, under serum-free conditions for 24 h to screen the optimal concentration of AST solution and the most effective concentration of IS solution. Then, cell viability was determined by 3-(4,5-dimethylthiazol-2-yl)-2,5-diphenyltetrazolium bromide (MTT) assay.

Based on viability screening in HK-2 cells, 20 μM AST and 250 μM IS were identified as the optimal working concentrations for subsequent experiments. The selected IS concentration is widely used *in vitro* to simulate uremic toxicity, and aligns with total plasma IS levels observed in advanced CKD patients (Park et al., 2019; Sun et al., 2025).

For studying the interactions between AST and IS, HK-2 cells were divided into four groups and subjected to different treatments: (1) the Control group: HK-2 cells were cultured under standard serum-free conditions without the addition of AST solution or IS solution; (2) the AST group: HK-2 cells were subjected to 20 μM of AST solution for 24 h under serum-free conditions; (3) the IS group: HK-2 cells received 250 μM of IS solution treatment for 24 h under serum-free conditions; (4) the IS + AST group: HK-2 cells were treated with 250 μM of IS solution and 20 μM of AST solution under serum-free conditions for 24 h.

### MTT assay

HK-2 cells, after pre-incubation, were inoculated in 96-well plates at a density of 5 × 10^3^ cells/well. Next, AST solution and/or IS solution were added for 24 h of incubation under serum-free conditions. After the incubation, the cells were washed with PBS buffer three times. Then, each well was supplemented with 20 μL of MTT solution (0.5 mg/mL, Beyotime, China) and incubated for 4 h at 37 °C. After that, the culture solution in the wells was aspirated and discarded. Subsequently, 150 μL of dimethyl sulfoxide (Beyotime, China) was added to each well, and the plates were placed on a shaker with low-speed shaking for 10 min to ensure that the crystals were fully dissolved. The absorbance value at 490 nm of each well was measured using a multimode microplate reader (Thermo Fisher Scientific, United States).

### Terminal deoxynucleotidyl transferase dUTP nick-end labeling (TUNEL) assay

The treated HK-2 cells were resuspended in PBS buffer, and 50 μL of cell suspension was dropped on the slide to prepare a cell smear. Subsequently, the cells were fixed with 4% paraformaldehyde for 30 min and washed with PBS buffer. Then, the slide was supplemented with 50 μL of terminal deoxynucleotidyl transferase dUTP nick-end labeling detection solution (Servicebio, China) slowly, followed by incubation at 37 °C for 60 min in the dark. After washing three times with PBS buffer, 4′,6-diamidino-2-phenylindole (#ab104139, Abcam, United Kingdom) was gently added to the slides and incubated at 37 °C for 10 min to stain the nuclei in the dark. Ultimately, five randomly selected fields of view of each sample were photographed using a fluorescence microscope (Olympus, Japan). The images obtained were used to quantitatively analyze the number of apoptotic cells using ImageJ software (National Institutes of Health, United States), and the average value was taken as the final result.

### Measurement of reactive oxygen species

In this study, the fluorescent probe CM-H2DCFDA (MedChemExpress, United States) was used to detect intracellular reactive oxygen species (ROS) levelS. Briefly, the treated HK-2 cell culture wells were supplemented with 10 μM of CM-H2DCFDA solution and incubated for 30 min at 37 °C in the dark. Each sample was photographed under a fluorescence microscope by randomly selecting 5 fields of view. The images obtained were quantitatively analyzed for the fluorescence intensity using ImageJ software to reflect the ROS level, and the average value was taken as the final result.

### Determination of glutathione, superoxide dismutase and malondialdehyde

The levels of intracellular oxidative stress indicators were determined, following the methods proposed in a previous study (Guo et al., 2023). The treated HK-2 cells from each group were washed with PBS buffer. Upon low-speed centrifugation, the cell pellets were collected and cells were resuspended in 0.5 mL PBS buffer. Then, cells were lysed with an ultrasonic cell pulverizer (Sonics, United States). Subsequently, the supernatant was collected for the determination of glutathione (GSH), superoxide dismutase (SOD), and malondialdehyde (MDA) levels. According to the instructions, the supernatant was supplemented with various reagents from the GSH Assay Kit (Servicebio, China), SOD Assay Kit (Servicebio, China) and MDA Assay Kit (Servicebio, China), respectively, for mixed incubation. Ultimately, the multimode microplate reader was used to measure the absorbance at 412 nm for the GSH level, 550 nm for the SOD level and 532 nm for the MDA level.

### Alkaline comet assay

The severity of DNA damage in the cells was assessed by the alkaline comet assay, and the experimental procedures were conducted in accordance with a previous study (Lu et al., 2017). The treated HK-2 cells were digested with trypsin and centrifuged to create a single-cell suspension. The single-cell suspension was mixed with 1% Low Melting Point Agarose at a ratio of 1:10. Then, 30 µL of the mixture was added dropwise onto a slide and allowed to stand for 10 min at 4 °C in the dark. Cells were lysed with lysis buffer and the DNA was unwound with fatty alcohol polyoxyethylene ether sodium sulfate. Subsequently, the slides were subjected to electrophoresis at 30 V and 400 mA for 30 min. After electrophoresis, 50 µL of red fluorescent nucleic acid stain (Solarbio, China) was added for 15 min of incubation at room temperature in the dark. Ultimately, five randomly selected fields of view of each sample were photographed under a fluorescence microscope, and the images were quantitatively analyzed using ImageJ software. After the comet tail DNA level was measured, the comet tail moment (TM) value was calculated using the formula $TM=\frac{Tail\;length\times Tail\%DNA}{100}$ , and the average value was taken as the final result.

### Senescence-associated beta-galactosidase (SA-β-gal) staining

In this study, the cellular senescence level was assessed by SA-β-gal staining. The staining fixative and staining working solutions were prepared in the SA-β-gal kit (Abcam, United Kingdom) according to the instructions. The treated HK-2 cells from each group were washed with PBS buffer and fixed with 1 mL of staining fixative solution for 15 min at room temperature. Upon fixation, the fixative solution was aspirated and discarded. Then, 1 mL of SA-β-gal staining working solution was added for incubation overnight at 37 °C in the dark. The following day, five random fields of each sample were selected and photographed under a light microscope (Olympus, Japan). The number of positively stained cells was quantitatively analyzed using ImageJ software, and the average value was taken as the final result.

### Western blot

Total intracellular proteins were extracted using radioimmunoprecipitation lysis buffer (Servicebio, China) containing protease inhibitors and phosphatase inhibitors. The protein concentrations were determined by a bicinchoninic acid protein assay kit (Abcam, United Kingdom). Upon separation by sodium dodecyl sulfate polyacrylamide gel electrophoresis (Servicebio, China), the total protein samples were transferred to the polyvinylidene fluoride (PVDF) membrane (Millipore, United States). The PVDF membrane was blocked with 5% skimmed milk for 1 h at room temperature. Subsequently, the PVDF membrane was incubated with primary antibodies overnight at 4 °C. The primary antibodies used were displayed as follows: Bax (1:1,000, #ab32503, Abcam, United Kingdom); PARP1 (1:1,000, #ab191217, Abcam, United Kingdom); Anti-Cleaved PARP1 (1:1,000, #ab32064, Abcam, United Kingdom); Caspase-3 (1:5000, #ab32351, Abcam, United Kingdom); Cleaved Caspase-3 (1:500, #ab32042, Abcam, United Kingdom); γH2AX (1:5,000, #ab81299, Abcam, United Kingdom); ataxia telangiectasia and Rad3-related protein (ATR, 1:1,000, #ab175221, Abcam, United Kingdom); phospho-ATR (p-ATR, 1:1,000, #ab316925, Abcam, United Kingdom); P53 (1:1,000, #ab26, Abcam, United Kingdom); P21 (1:1,000, #ab109520, Abcam, United Kingdom); Kelch-like ECH-associated protein 1 (KEAP1, 1:4,000, #ab139729, Abcam, United Kingdom); NAD (P)H quinone dehydrogenase 1 (NQO1, 1:20,000, #ab80588, Abcam, United Kingdom); heme oxygenase-1 (HO-1, 1:2,000, #ab189491, Abcam, United Kingdom); nuclear factor erythroid 2-related factor 2 (Nrf2, 1:1,000, #ab313825, Abcam, United Kingdom); glyceraldehyde-3-phosphate dehydrogenase (GAPDH, 1:2,500, #ab9485, Abcam, United Kingdom); H3 (1:5,000, #ab1791, Abcam, United Kingdom). The next day, after rinsing with tris buffered saline with tween buffer, the PVDF membranes were incubated with horseradish peroxidase-labeled Goat Anti-Rabbit IgG (H + L) secondary antibody (1:5,000, #ab6721, Abcam, United Kingdom) for 1 h at room temperature. Ultimately, the membranes were visualized using a gel imaging analysis system (Tanon, China) and the expression level of each protein was quantitatively analyzed by ImageJ software. The expression levels of glyceraldehyde-3-phosphate dehydrogenase and H3 proteins served as internal references to correct the expression levels of each target protein.

### Statistical analysis

The obtained data were statistically analyzed using SPSS 22.0 software (IBM, United States). Normality of the data distribution was verified using the Shapiro–Wilk test. The homogeneity of variances was confirmed using Levene’s test. All data were expressed as mean ± standard deviation (SD) from three independent experiments (n = 3). Differences among multiple groups were assessed using one-way analysis of variance (ANOVA), followed by Tukey’s *post hoc* test for multiple comparisons. *P* < 0.05 was considered statistically significant.

## Results

### Identification of candidate targets of AST in uremia

**FIGURE 1:**
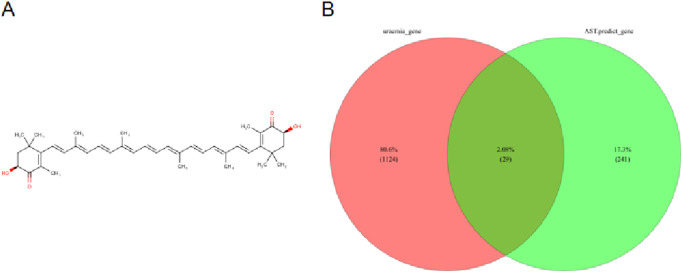
Identification of candidate targets of AST. **(A)** Chemical structure of AST retrieved from the PubChem database. **(B)** Venn diagram showing the overlap between AST-predicted targets (n = 270) and uremia-related genes (n = 1,153).

To identify potential targets of AST against uremia-related injury, the chemical structure of AST was first retrieved from the PubChem database (Figure 1A). AST-related targets (289 in total: 27 from SwissTargetPrediction, 251 from PharmMapper, 11 from SEA) were merged and de-duplicated to yield 270 unique targets, while 1,153 uremia-related genes were obtained from GeneCards, OMIM, and GEO datasets, including 1,033 DEGs from GSE37171 (74 uremia vs. 41 healthy) under |log2FC|>1 and adjusted *P* < 0.05. By intersecting 270 AST-related targets with 1,153 CKD/uremia-related genes, 29 overlapping genes were identified as candidate targets (Figure 1B).

### Functional enrichment analysis and identification of hub genes

**FIGURE 2:**
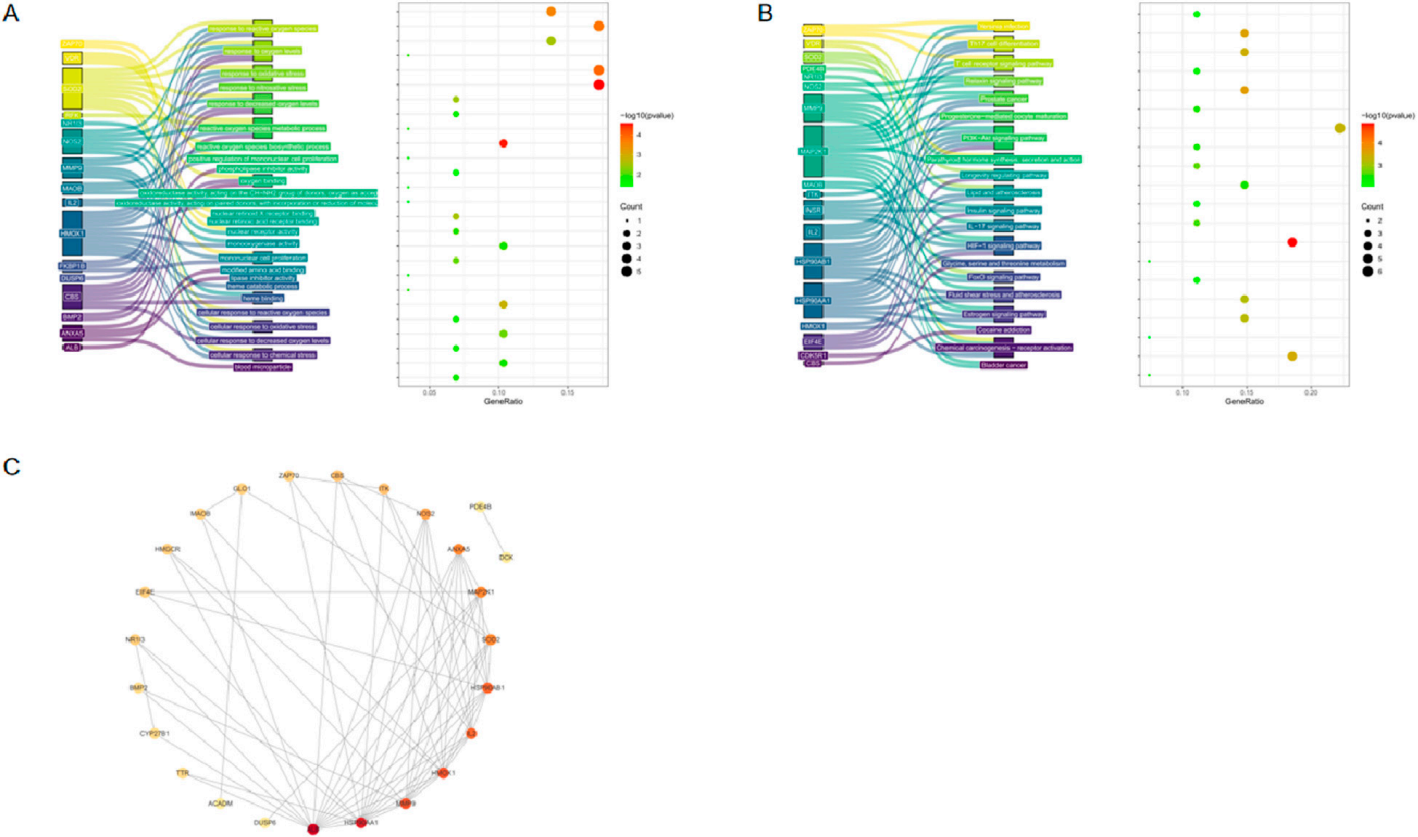
Functional enrichment and PPI network analysis of candidate targets. **(A)** GO enrichment analysis of candidate targets, showing significant enrichment in oxidative stress-related biological processes. **(B)** KEGG pathway enrichment analysis, indicating involvement in oxidative stress- and inflammation-related signaling pathways. **(C)** Protein–protein interaction (PPI) network of 29 candidate targets constructed using the STRING database.

To further elucidate the biological roles of the 29 candidate targets, functional enrichment analysis was conducted. GO enrichment yielded 559 significant terms (adjusted *P* < 0.05), mainly involving oxidative stress–related processes such as cellular response to oxidative stress, heme binding, and reactive oxygen species metabolic processes (Figure 2A). Keyword filtering further highlighted 26 GO terms related to oxidative stress and HO-1 (Figure 2A). KEGG pathway analysis identified 42 significantly enriched pathways (adjusted *P* < 0.05). The top-ranked pathways included the PI3K-Akt signaling pathway and MAPK signaling pathway, which are known master regulators of cellular processes including survival, proliferation, and stress response. Although a direct keyword search for oxidative stress or HO-1 did not yield a specific hit, the enrichment of these broad signaling hubs is highly consistent with the oxidative stress-related GO terms. This suggests that the antioxidant response is orchestrated through these central, multi-functional signaling networks rather than a single, isolated pathway (Figure 2B). In addition, a PPI network was constructed using the STRING database. The PPI network highlighted 10 hub genes: ALB, HSP90AA1, MMP9, HSP90AB1, ANXA5, HMOX1, IL2, MAP2K1, SOD2, and NOS2 (Figure 2C).

### Construction of the drug–target–pathway network

**FIGURE 3:**
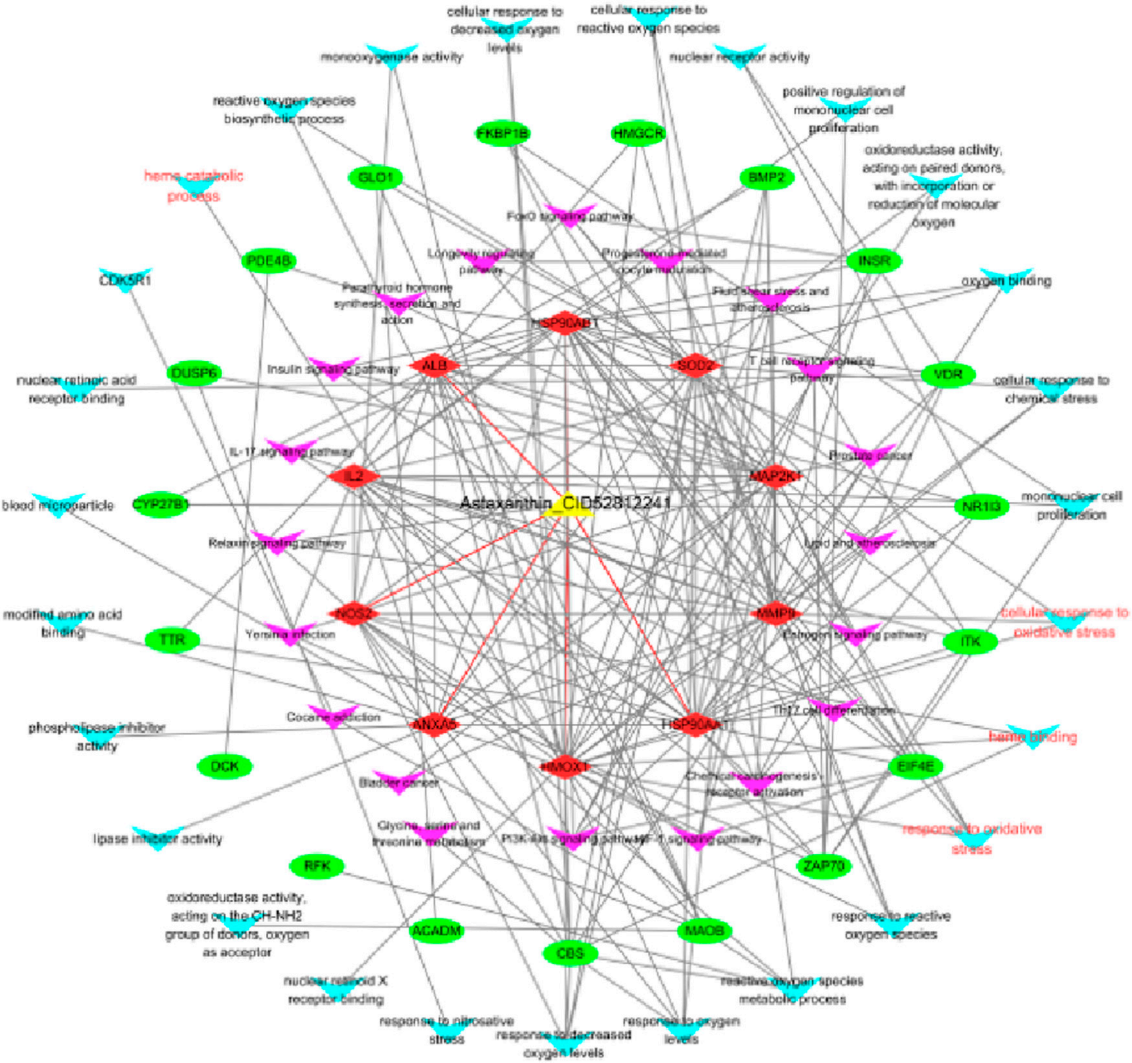
Construction of the drug–target–pathway network. The network illustrates the interactions among AST (yellow triangle), candidate targets (red and green nodes), GO pathways (purple nodes), and KEGG pathways (sky-blue nodes).

To visualize the complex interactions between AST, candidate targets, and enriched pathways, a drug–target–pathway network was constructed (Figure 3). The network used the following legend: AST = yellow triangle; hub genes = red diamonds; other targets = green ovals; GO terms = purple V-shapes; KEGG terms = sky-blue V-shapes; red edges = stable docking interactions; oxidative stress/HO-1 terms labeled in red. Hub targets such as HMOX1, SOD2, NOS2, and ALB showed strong connectivity with oxidative stress-related pathways, including cellular response to reactive oxygen species and heme catabolic process.

### Molecular docking validation of hub targets

Molecular docking analysis was performed to evaluate the binding affinity between AST and hub proteins. AST exhibited stable binding with several hub targets (Table 1). Among them, NOS2 (−7.6 kcal/mol), ANXA5 (−7.5 kcal/mol), and ALB (−7.2 kcal/mol) demonstrated the strongest interactions, followed by HSP90AA1 (−6.8 kcal/mol) and HMOX1 (−6.9 kcal/mol). Notably, HMOX1 and SOD2, which are closely related to oxidative stress and the Nrf2/HO-1 signaling pathway, also showed favorable binding affinities with AST.

**TABLE 1:** Binding energies of AST with hub gene proteins.

1	1	−7.2	0	0	ALB_1ao6
10	1	−7.5	0	0	ANXA5_1anw
19	1	−6.9	0	0	HMOX1_1n45
28	1	−6.8	0	0	HSP90AA1_1byq
37	1	−7.2	0	0	HSP90AB1_1qz2
46	1	−6.5	0	0	IL2_1m47
55	1	−6.9	0	0	MAP2K1_1s9j
64	1	−6.2	0	0	MMP9_1gkc
73	1	−7.6	0	0	NOS2_1nsi
82	1	−6.4	0	0	SOD2_1ap5

### Molecular docking visualization of hub targets

**FIGURE 4:**
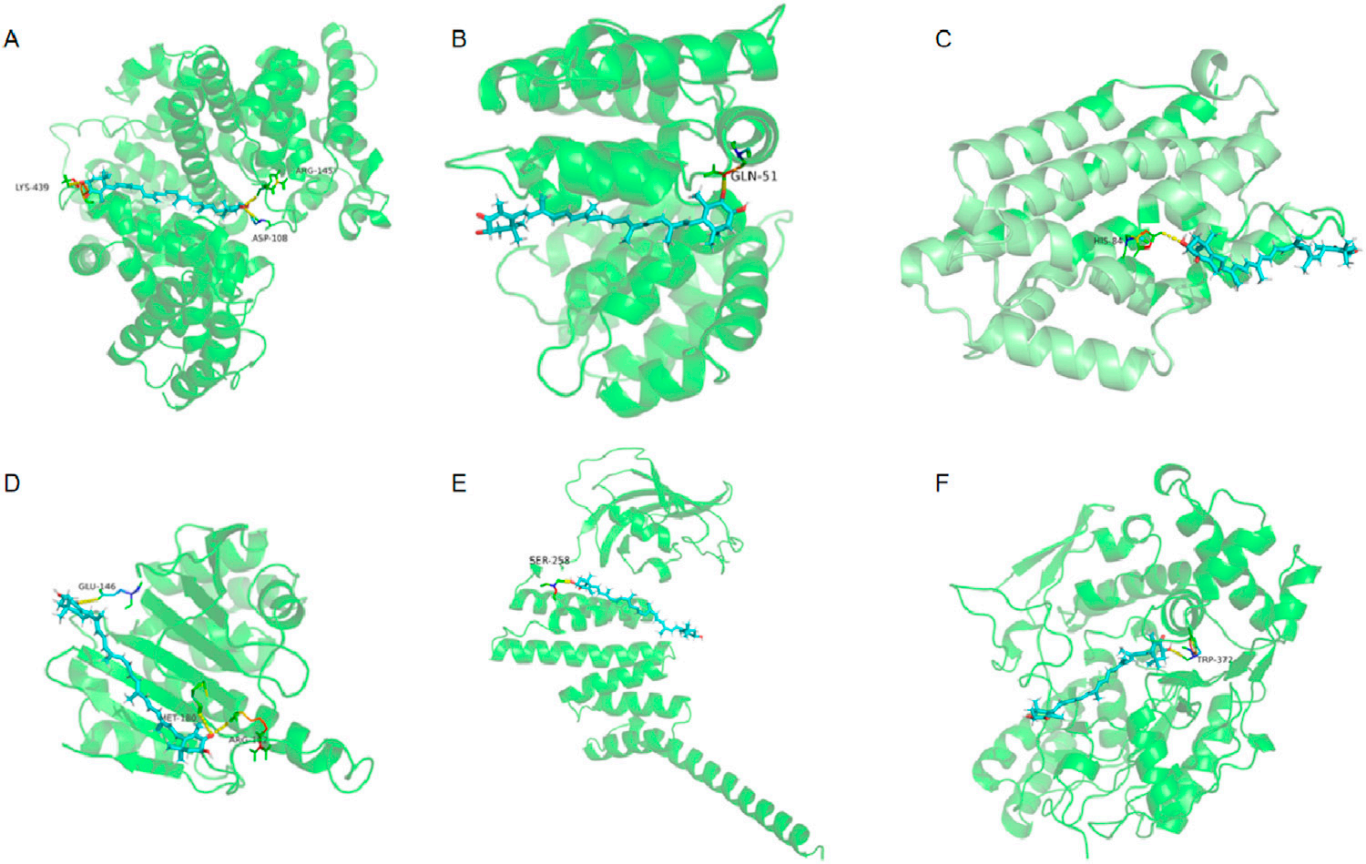
Molecular docking of AST with hub proteins. **(A)** Docking conformation of AST with ALB, showing hydrogen bonds with Lys439, Arg145, and Asp108. **(B)** Docking conformation of AST with ANXA5, showing interaction with Gln51. **(C)** Docking conformation of AST with HMOX1, showing hydrogen bonding with His84. **(D)** Docking conformation of AST with HSP90AA1, showing interactions with Met180, Glu146, and Arg32. **(E)** Docking conformation of AST with HSP90AB1, showing interaction with Ser258. **(F)** Docking conformation of AST with NOS2, showing interaction with Trp372. Green ribbon structures represent proteins, blue sticks represent AST molecules, and yellow dashed lines indicate hydrogen bonds.

The binding conformations of AST with hub proteins were further visualized to confirm the molecular interactions (Figure 4). AST formed stable hydrogen bonds with several key residues within the binding pockets of the target proteins. Specifically, hydrogen bond interactions were observed between AST and ALB (Lys439, Arg145, Asp108), ANXA5 (Gln51), HMOX1 (His84), HSP90AA1 (Met180, Glu146, Arg32), HSP90AB1 (Ser258), and NOS2 (Trp372).

### Screening of IS and AST dosing concentrations

**FIGURE 5:**
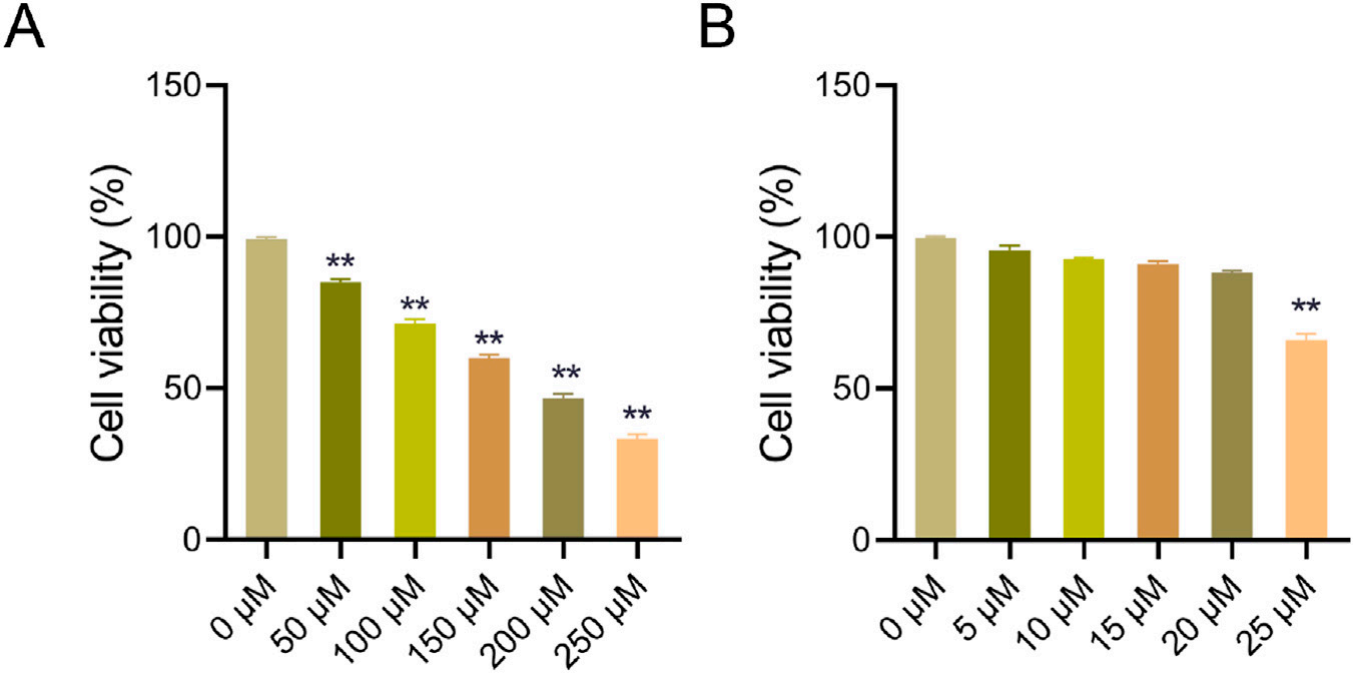
Toxicity of indoxyl sulfate and astaxanthin on HK-2 cells. **(A)** MTT assay was used to detect the viability of HK-2 cells exposed to 0 μM, 50 μM, 100 μM, 150 μM, 200 μM, and 250 μM IS solution. **(B)** MTT assay was utilized to measure the viability of HK-2 cells treated with 0 μM, 5 μM, 10 μM, 15 μM, 20 μM, and 25 μM AST solution. Data were expressed as mean ± SD (n = 3). ^**^ *P* < 0.01 vs. 0 μM. MTT, 3-(4,5-dimethylthiazol-2-yl)-2,5-diphenyltetrazolium bromide; IS, indoxyl sulfate; AST, astaxanthin.

Firstly, we prepared six different concentration gradients of IS solution and AST solution to observe the toxicity of IS and AST on HK-2 cells respectively, thereby selecting the appropriate concentrations of IS and AST for subsequent studies. The results of the MTT experiments showed that after treatment with IS solution alone on HK-2 cells, the viability of HK-2 cells was significantly decreased in the 50 μM, 100 μM, 150 μM, 200 μM, and 250 μM groups compared with the 0 μM group (*P* < 0.05), in a concentration-dependent manner (Figure 5A). In addition, after treatment with AST solution alone on HK-2 cells, there was no significant difference in the HK-2 cell viability in the 0 μM, 5 μM, 10 μM, 15 μM, and 20 μM groups (*P* > 0.05); and HK-2 cell viability in the 25 μM group was markedly reduced (*P* < 0.05) as opposed to the 0 μM group (Figure 5B). As this study proposed to use IS to damage renal tubular epithelial cells to construct an *in vitro* CKD model, we selected the 250 μM IS solution with the greatest cytotoxicity (i.e., the lowest viability of HK-2 cells) on HK-2 cells, for subsequent experiments. Additionally, to explore the renal protective effect of AST, we selected the 20 μM AST solution with higher concentration but less cytotoxicity, for subsequent experiments.

### AST increases the viability and inhibits apoptosis of HK-2 cells exposed to IS

Renal tubular epithelial cells are responsible for the reabsorption of water, electrolytes, and metabolic wastes. If their numbers are insufficient, the kidneys cannot maintain normal function, thereby leading to a sustained decline in renal function (Evans et al., 2022). In an *in vitro* model, the effect of AST on the number of renal tubular cells was analyzed by measuring the viability and apoptosis of HK-2 cells.

**FIGURE 6:**
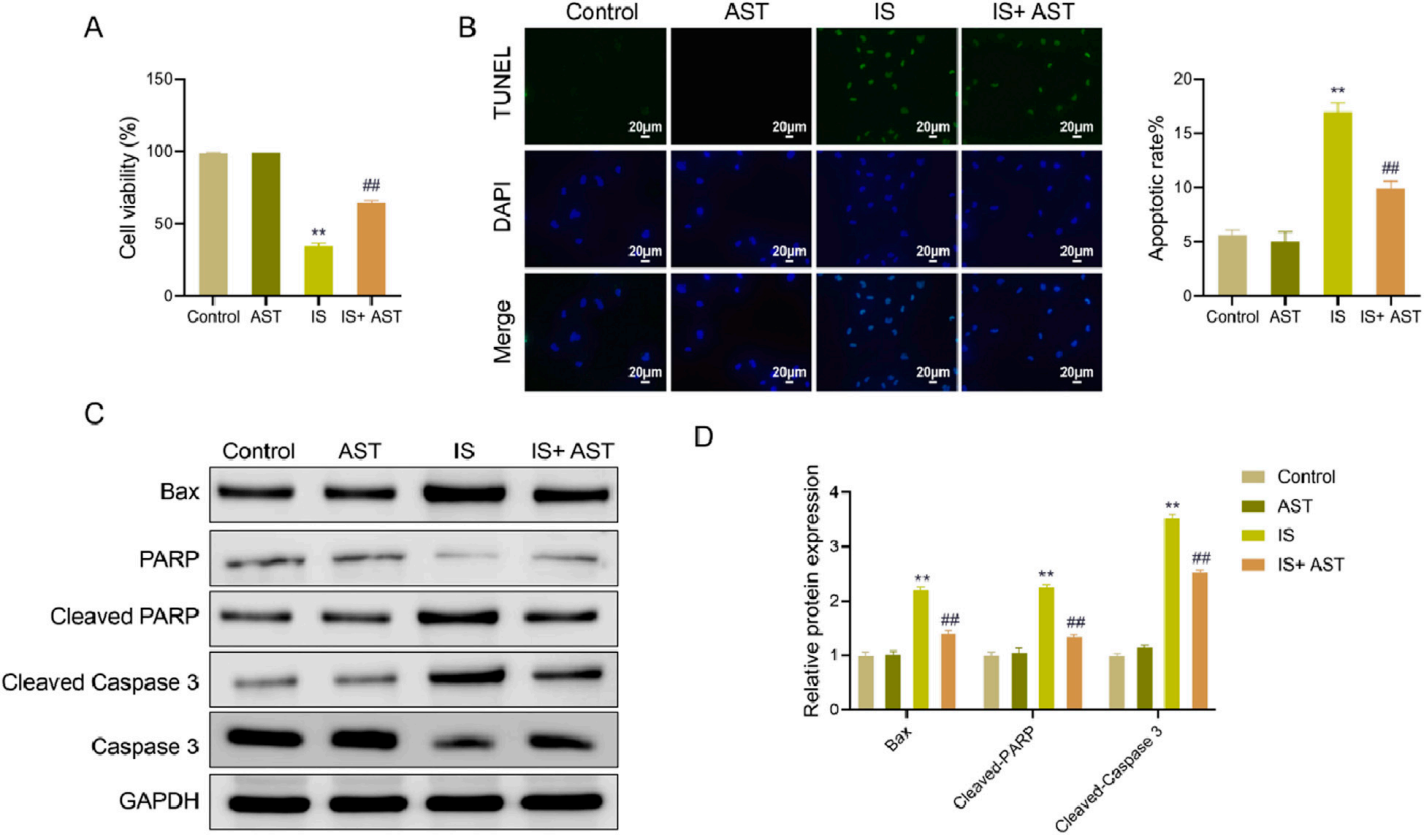
AST increases the viability and inhibits apoptosis of HK-2 cells exposed to IS. **(A,B)** MTT assay and TUNEL assay were used to determine the viability and apoptosis of HK-2 cells in different treatment groups, respectively. **(C)** Western blot was employed to measure the protein expression levels of Bax, cleaved-PARP, PARP, caspase-3, and cleaved caspase-3 in HK-2 cells in different treatment groups. **(D)** The protein expression levels of Bax, cleaved-PARP, and cleaved caspase-3 were quantitatively analyzed. Data were expressed as mean ± SD (n = 3). ^**^ *P* < 0.01 vs. Control; ^##^ *P* < 0.01 vs. IS. TUNEL, terminal deoxynucleotidyl transferase dUTP nick-end labeling; Bax, bcl-2-associated X protein; cleaved-PARP, cleaved poly (ADP-ribose) polymerase.

Regarding cell viability, the results of MTT experiments displayed that relative to the Control group, the HK-2 cell viability was significantly decreased in the IS group (*P* < 0.05). Compared to the IS group, the HK-2 cell viability was markedly increased in the IS + AST group (*P* < 0.05). Cell viability was slightly higher in the AST group than that in the Control group, but the difference was not statistically significant (*P* > 0.05) (Figure 6A).

For apoptosis, the terminal deoxynucleotidyl transferase dUTP nick-end labeling staining results revealed that the apoptosis level of HK-2 cells was significantly higher in the IS group than that in the Control group (*P* < 0.05). As opposed to the IS group, there was a notable reduction in the apoptosis level of HK-2 cells in the IS + AST group (*P* < 0.05). The apoptosis level of cells was slightly lower in the AST group in comparison with the Control group, but no statistically significant difference was observed (*P* > 0.05) (Figure 6B). In addition, Western blot was employed to detect the levels of several apoptosis-related proteins. The findings displayed that in contrast to the Control group, the IS group exhibited an upward trend in the expression levels of Bax, cleaved PPAR, and cleaved caspase-3 (*P* < 0.05). Compared to the IS group, the above protein expression was markedly declined in the IS + AST group (*P* < 0.05). No significant change was observed in the AST group compared with the Control group (*P* > 0.05) (Figures 6C,D).

These results indicated that AST significantly increased the viability and inhibited the apoptosis of HK-2 cells after IS treatment. This suggested that AST had an effect on maintaining the number of renal tubular epithelial cells, thus confirming the renal protective effect of AST in the IS environment.

### AST attenuates oxidative stress in HK-2 cells exposed to IS

Oxidative stress is a significant driving factor in CKD progression, and alleviating oxidative stress is considered an effective strategy to slow CKD progression (Daenen et al., 2019). Therefore, the oxidative stress-related indicators were further examined to explore whether AST enhances HK-2 cell viability and inhibits apoptosis by modulating oxidative stress.

**FIGURE 7:**
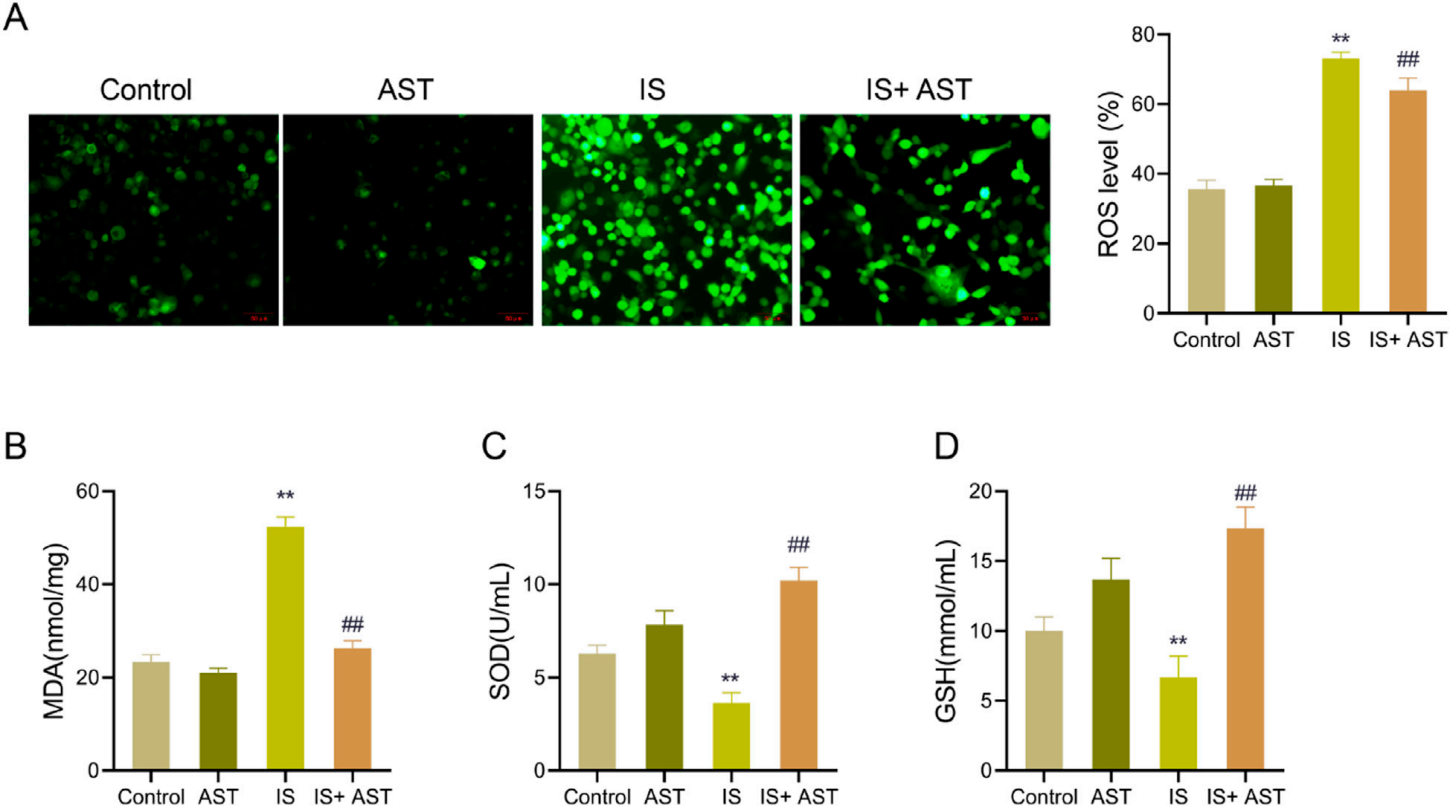
AST attenuates the severity of IS-induced oxidative stress in HK-2 cells. **(A)** The fluorescence probe (CM-H2DCFDA) was used to detect the ROS levels in HK-2 cells in the Control group, the AST group, the IS group, and the IS + AST group. **(B–D)** Kits were used to measure the levels of MDA, SOD, and GSH in HK-2 cells of different groups. Data were expressed as mean ± SD (n = 3). ^**^ *P* < 0.01 vs. Control; ^##^ *P* < 0.01 vs. IS. ROS, reactive oxygen species; MDA, malondialdehyde; SOD, superoxide dismutase; GSH, glutathione.

The fluorescent probe results presented that the ROS level in HK-2 cells was significantly increased in the IS group compared with the Control group (*P* < 0.05); the ROS level in HK-2 cells was markedly decreased in the IS + AST group as opposed to the IS group (*P* < 0.05); and the ROS level was lower in the AST group than that in the Control group (*P* > 0.05), with no statistically significant difference (Figure 7A). In addition, relative to the Control group, there was a notable elevation in the level of MDA in the IS group (*P* < 0.05), and a remarkable reduction in the levels of SOD and GSH (*P* < 0.05). As opposed to the IS group, the level of MDA was considerably downregulated in the IS + AST group (*P* < 0.05), while the levels of SOD and GSH were significantly raised (*P* < 0.05). The levels of ROS were lower in the AST group than those in the Control group, but the levels of SOD and GSH were higher. The differences were not statistically significant (*P* > 0.05) (Figures 7B–D). The above data suggested that AST could effectively reduce the severity of oxidative stress in HK-2 cells exposed to IS.

### AST ameliorates DNA damage and cellular senescence in HK-2 cells exposed to IS

DNA damage and senescent phenotype of renal tubular epithelial cells are distinctive features of CKD progression (Wang et al., 2017). This finding suggests that inhibition of DNA damage and cellular senescence processes is essential for kidney protection. Therefore, we examined the relevant indicators of DNA damage and cellular senescence.

**FIGURE 8:**
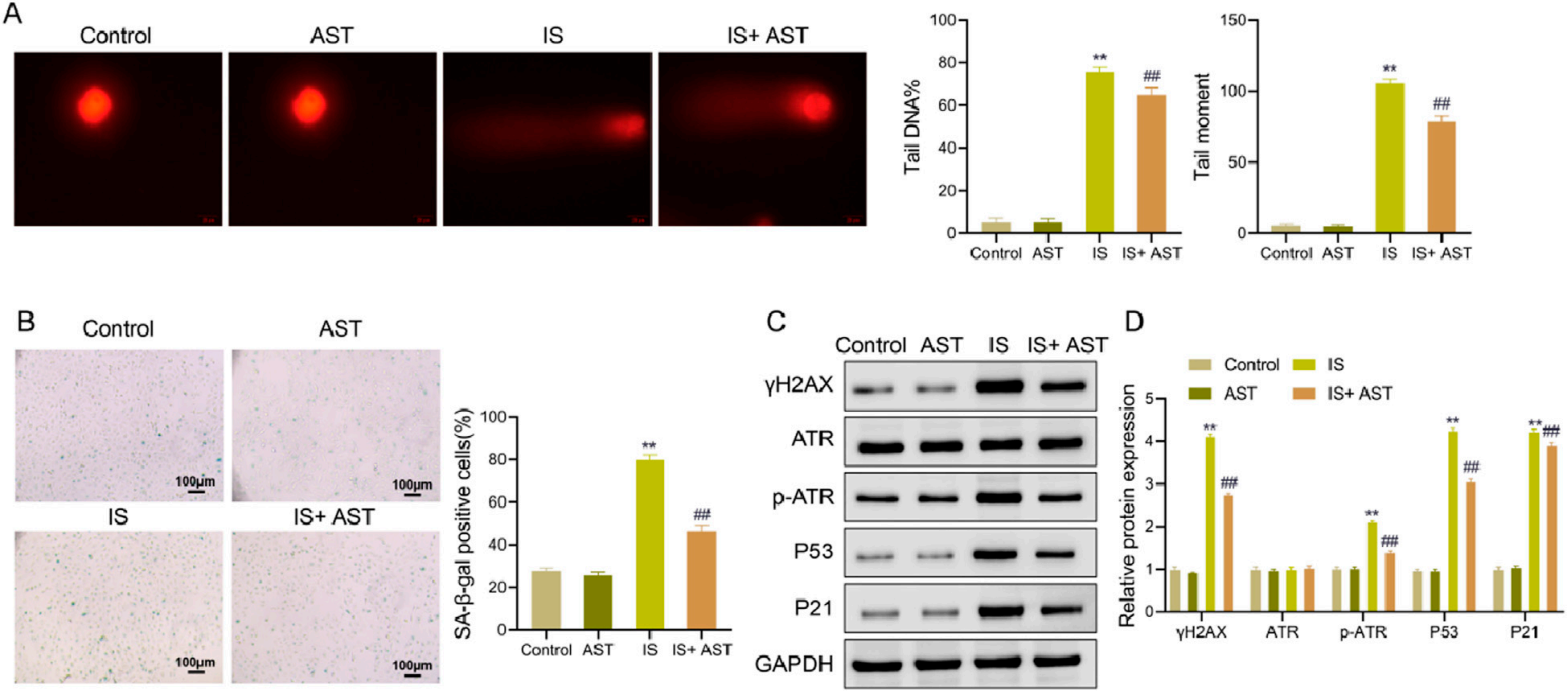
AST ameliorates DNA damage and cellular senescence in HK-2 cells exposed to IS. **(A)** Alkaline comet assay was used to detect the comet tail DNA percentage and comet TM of HK-2 cells in different treatment groups. **(B,C)** SA-β-gal staining was utilized to assess the senescence level of HK-2 cells in different treatment groups. **(D)** Western blot was performed to detect the protein expression levels of γH2AX, ATR, p-ATR, P53, and P21 of HK-2 cells in different treatment groups; E, The protein expression levels of γH2AX, ATR, p-ATR, P53, and P21 were quantitatively analyzed. Data were expressed as mean ± SD (n = 3). ^**^ *P* < 0.01 vs. Control; ^##^ *P* < 0.01 vs. IS. TM, tail moment; SA-β-gal, senescence-associated beta-galactosidase; ATR, ataxia telangiectasia and Rad3-related protein; p-ATR, phospho-ATR.

Regarding DNA damage, the results of the alkaline comet assay showed that compared with the Control group, there was a significant increase in the DNA damage level (*P* < 0.05), the comet tail DNA percentage, and comet TM (*P* < 0.05) in the IS group. In contrast to the IS group, the IS + AST group exhibited a marked reduction in the DNA damage level (*P* < 0.05), the comet tail DNA percentage and comet TM (*P* < 0.05). Additionally, the above indicators were higher in the AST group than those in the Control group, with no statistically significant differences (*P* > 0.05) (Figure 8A). The results of the Western blot demonstrated that compared with the Control group, the expression level of γH2AX protein was markedly increased in the IS group (*P* < 0.05). In comparison with the IS group, the expression level of γH2AX protein was remarkably decreased in the IS + AST group (*P* < 0.05); There was no significant difference between the Control and AST groups (*P* > 0.05) (Figure 8D).

For cellular senescence, the SA-β-gal staining results displayed that the number of blue positive cells was significantly increased compared with the Control group (*P* < 0.05). Relative to the IS group, the IS + AST group presented a notable reduction in the number of blue positive cells (*P* < 0.05). The number of blue positive cells was lower in the AST group than that in the Control group, with no statistically significant differences (*P* > 0.05) (Figures 8B,C). Western blot was used to detect the levels of key cell cycle regulatory proteins, as cellular senescence is often accompanied by cell cycle arrest The results exhibited that in comparison with the Control group, there was a remarkable upregulation in the protein expression levels of p-ATR, P53, and P21 in the IS group (*P* < 0.05), and there was no significant difference in the protein levels of ATR (*P* > 0.05). The levels of p-ATR, P53, and P21 protein expression were considerably downregulated in the IS + AST group in contrast to the IS group (*P* < 0.05), and no significant difference was observed in the protein levels of ATR (*P* > 0.05). There was no statistically significant difference between the Control and AST groups (*P* > 0.05) (Figure 8D).

Overall, these results indicate that AST significantly ameliorates DNA damage and cellular senescence in HK-2 cells exposed to IS.

### AST activates the Nrf2/HO-1 pathway in HK-2 cells exposed to IS

The Nrf2/HO-1 pathway plays a key antioxidant role *in vivo* (Loboda et al., 2016). Therefore, we further explored whether the therapeutic effects of AST on CKD are related to this pathway.

**FIGURE 9:**
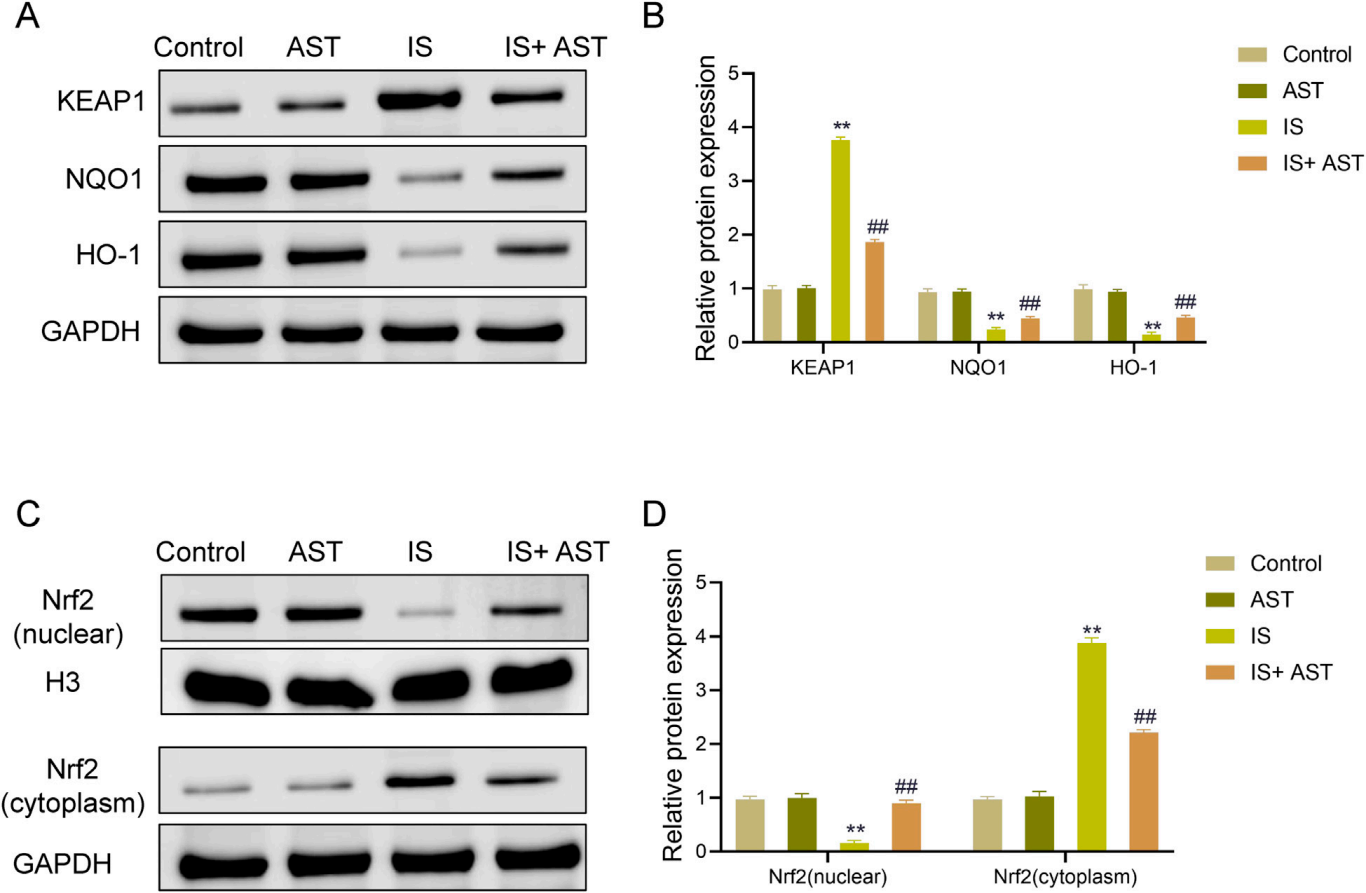
AST activates the Nrf2/HO-1 pathway in IS-exposed HK-2 cells. **(A,B)** Western blot was adopted to detect the protein expression levels of KEAP1, NQO1, and HO-1 in different groups. **(C,D)** Western blot was applied to determine the expression levels of Nrf2 in the cytoplasm and nucleus in different groups. Data were expressed as mean ± SD (n = 3). ^**^ *P* < 0.01 vs. Control; ^##^ *P* < 0.01 vs. IS. KEAP1, Kelch-like ECH-associated protein 1; NQO1, NAD (P)H quinone dehydrogenase 1; HO-1, heme oxygenase-1; Nrf2, nuclear factor erythroid 2-related factor 2.

The results of the Western blot showed that the protein level of KEAP1 was significantly raised in the IS group (*P* < 0.05), while the protein levels of NQO1 and HO-1 were markedly reduced (*P* < 0.05) compared with the Control group. Relative to the IS group, the IS + AST group displayed a notable downregulation in the protein level of KEAP1 (*P* < 0.05) and a remarkable upregulation in the protein levels of NQO1 and HO-1 (*P* < 0.05). There were no statistically significant differences between the Control and AST groups (*P* > 0.05) (Figures 9A,B). As opposed to the Control group, the expression level of Nrf2 in the nucleus was considerably declined in the IS group (*P* < 0.05), and that in the cytoplasm was markedly raised (*P* < 0.05). In contrast to the IS group, the expression level of Nrf2 was significantly elevated in the nucleus in the IS + AST group (*P* < 0.05), but was reduced in the cytoplasm (*P* < 0.05). There was no statistically significant difference between the Control and AST groups (*P* > 0.05) (Figures 9C,D). These results suggested that AST could significantly activate the Nrf2/HO-1 pathway in HK-2 cells exposed to IS.

## Discussion

This study combined network pharmacology with experimental verification to elucidate the protective effect of AST on renal tubular injury induced by IS. The main mechanism might be through activating the Nrf2/HO-1 signaling pathway. Computational analysis revealed 29 overlapping targets enriched in the oxidative stress pathway. Molecular docking experiments confirmed stable binding with hub proteins such as HMOX1 and SOD2. The results of *in vitro* experiments consistently showed that AST effectively alleviated IS-induced HK-2 cell injury by increasing cell survival rate, inhibiting cell apoptosis, reducing oxidative stress, decreasing DNA damage, and delaying cell aging. These findings indicate that AST plays multiple renal protective roles in CKD.

Our network pharmacology analysis indicates that oxidative stress regulation is the core mechanism by which AST exerts its renal protective effect in chronic kidney disease, and the Nrf2/HO-1 axis becomes the key pathway of this mechanism. Among them, HMOX1, SOD2, and NOS2 have become the key targets - which is consistent with their previous research that can protect renal tubules from oxidative damage, maintain mitochondrial antioxidant capacity, and regulate the oxidative-reductive imbalance associated with uremia (Kitada et al., 2020; Li et al., 2021). Although previous studies have reported the antioxidant properties of AST in various kidney disease models (Xie et al., 2018), our work integrates computational and experimental methods to systematically link these effects with specific hub gene systems, thereby expanding these findings. Moreover, KEGG enrichment analysis reveals a broader upstream signaling network, including PI3K-Akt and MAPK, which are closely related to regulating the Nrf2/KEAP1 axis. Therefore, AST does not act through a single “oxidative stress pathway”, but regulates multiple interrelated oxidative stress-related nodes. This is not contradictory to the oxidative stress focus revealed by GO analysis, but rather provides a reasonable upstream mechanism framework for it, highlighting the multi-target and network pharmacology characteristics of AST.

The human renal tubular epithelial cell line HK-2 was selected as the *in vitro* model because it plays a crucial role in maintaining electrolyte and fluid balance, and is susceptible to the progression of CKD (Vallon, 2011; Chevalier, 2016). IS is a protein-bound uremic toxin derived from the metabolism of tryptophan in the intestine, which accumulates in CKD patients and has been proven to disrupt mitochondrial function and promote epithelial-mesenchymal transition of renal cells (Cai et al., 2018; Cai et al., 2021). Therefore, we chose to treat HK-2 cells with IS to simulate the characteristics of kidney damage caused by CKD. In this study, IS significantly reduced cell viability and induced apoptosis, which confirmed its suitability for simulating the renal tubular damage associated with CKD. The selected 250 μM IS concentration not only significantly reduced cell viability but also could strongly reproduce the key features of CKD, including oxidative stress, DNA damage, and cell senescence, thereby providing a relevant damage environment for treatment evaluation. The choice of AST was based on its previously reported renal protective effect in models of renal fibrosis (Diao et al., 2019), acute kidney injury (Liu et al., 2018), and diabetic nephropathy (Naito et al., 2021). As a liposoluble carotenoid, AST has good safety characteristics and a minimal renal excretion burden (Nishida et al., 2023; Bohn et al., 2017; Baker and Perazella, 2020). In our experiments, 20 μM of AST did not show cytotoxicity but exhibited significant pharmacological activity, capable of significantly restoring the Nrf2/HO-1 signaling pathway and reversing the damage caused by IS. These results support the dose selection and highlight the selective protective effect of AST under pathological stress conditions.

To deeply analyze the protective mechanism of AST against ischemic renal injury, we focused on two key and interrelated pathological processes in CKD: oxidative stress and cell senescence accompanied by DNA damage. The results showed that AST directly countered the main pathological processes triggered by IS. IS significantly increased the levels of reactive oxygen species (ROS) and malondialdehyde (MDA), while inhibiting antioxidant defense mechanisms such as superoxide dismutase (SOD) and glutathione (GSH), which is consistent with oxidative stress as a key driver of chronic kidney disease (CKD) progression (Daenen et al., 2019). Meanwhile, AST treatment effectively restored the redox balance, which is consistent with its recognized antioxidant effect in cardiovascular diseases, cancer, and vascular aging models (Bansilal et al., 2015; Saini et al., 2020; Li et al., 2020). Additionally, IS caused severe genomic damage - manifested by the increase of γH2AX and the lengthening of comet tails - and promoted cell senescence by activating the ATR-p53-p21 axis, forming a harmful cycle that exacerbates renal tubular dysfunction (Wang et al., 2017; Kumari and Jat, 2021). AST significantly reduced DNA damage and senescence markers, promoting cell cycle re-entry and functional recovery. This is consistent with previous studies showing its ability to improve oxidative DNA damage and inhibit cell senescence in the lungs, skin, and vascular systems (Tang et al., 2023; Chung et al., 2018; Chao et al., 2020). Our research further indicates that AST simultaneously alleviated DNA damage and cell senescence in renal tubular cells damaged by IS, highlighting its multi-targeted cellular protective potential in chronic kidney disease. Although AST simultaneously improved apoptosis, oxidative stress, and senescence conditions, inhibiting senescence seems to be closely related to its genomic protection and redox regulatory functions, rather than merely a secondary result of reduced apoptosis. Further studies using gene intervention methods (such as p53, ATR, or cell cycle checkpoint inhibition) are necessary to reveal the causal hierarchical relationship among these interrelated injury responses.

To further investigate the signaling mechanisms underlying AST-mediated protection in CKD, we focused on the Nrf2/HO-1 pathway, a central regulator of cellular antioxidant defense. Nrf2 is a redox-sensitive transcription factor that, under homeostatic conditions, is sequestered in the cytoplasm by Keap1 and targeted for proteasomal degradation. Upon oxidative stress, Keap1 is inactivated, allowing Nrf2 to translocate into the nucleus, where it upregulates downstream antioxidant genes such as HO-1 and NQO1, thereby restoring redox balance (Guo et al., 2023; Satta et al., 2017). In this study, after AST intervention, the cytoplasmic Nrf2 decreased while nuclear Nrf2 increased, accompanied by upregulation of HO-1 and NQO1, indicating activation of the Nrf2/HO-1 pathway. These results suggested that the Nrf2/HO-1 pathway was activated by AST. Besides, AST can alleviate lung injury and liver injury by activating the Nrf2/HO-1 pathway (Luo et al., 2022; Cai et al., 2022), which aligns with the findings of this study. However, it remains possible that other regulatory pathways—such as PI3K/Akt, MAPK, or NF-κB—may also contribute to the observed protective effects of AST. Further investigation is warranted to fully elucidate the broader signaling network involved in its renoprotective actions.

Although our research has demonstrated that AST has the ability to restore Nrf2 nuclear translocation and upregulate the expression of HO-1/NQO1, the causal role of Nrf2 has not yet been directly verified through gene silencing techniques, which represents a key limitation. Future studies using siRNA or CRISPR-based methods will be crucial to confirm its necessity in the protective effect mediated by AST. Interestingly, IS exposure increased KEAP1 protein levels, a pattern that differs from the canonical model in which oxidative stress mainly inactivates KEAP1 via cysteine modification rather than elevating its abundance. However, recent studies show that KEAP1 can be upregulated under chronic pathological stress. Uremic toxins and persistent ROS can activate NF-κB, MAPK, and other stress-responsive pathways that enhance KEAP1 transcription (Xiao et al., 2024; Bellezza et al., 2018), while impaired autophagy may reduce its p62/SQSTM1-mediated degradation (Komatsu et al., 2010). Such non-canonical regulation likely explains the KEAP1 elevation observed in IS-treated cells and may further suppress Nrf2 activity, exacerbating oxidative vulnerability. The ability of AST to reverse KEAP1 upregulation therefore supports its role in restoring Nrf2 signaling under uremic toxin–induced stress. In addition to this mechanistic limitation, this study relies on a single renal epithelial cell line, the selective efficacy of AST only under pathological IS conditions, and concentration-dependent cytotoxicity at higher doses, all of which highlight the necessity for further validation. Future research that can explore Nrf2 functionally deficient models, more types of renal cells, and chronic kidney disease models *in vivo* (especially those that simulate IS accumulation) will be significant in confirming the therapeutic significance of AST and determining whether its protective effect on the kidney is applicable in different types of chronic kidney disease etiologies.

## Conclusion

This study is the first to demonstrate the therapeutic potential of AST in an IS-induced *in vitro* CKD model. Specifically, AST has the kidney protective effects of attenuating oxidative stress, ameliorating DNA damage and reversing cellular senescence, which may be related to its ability to activate the Nrf2/HO-1 pathway. This study provides new insights for the clinical treatment of CKD.

## Data Availability

The datasets presented in this study can be found in online repositories. The names of the repository/repositories and accession number(s) can be found in the article/supplementary material.

## References

[B1] Baker M. Perazella M. A. (2020). NSAIDs in CKD: are they safe? Am. J. Kidney Dis. 76 (4), 546–557. 10.1053/j.ajkd.2020.03.023 32479922

[B2] Bansilal S. Castellano J. M. Fuster V. (2015). Global burden of CVD: focus on secondary prevention of cardiovascular disease. Int. J. Cardiol. 201 (Suppl. 1), S1–S7. 10.1016/S0167-5273(15)31026-3 26747389

[B3] Bellezza I. Giambanco I. Minelli A. Donato R. (2018). Nrf2-Keap1 signaling in oxidative and reductive stress. Biochim. Biophys. Acta Mol. Cell Res. 1865 (5), 721–733. 10.1016/j.bbamcr.2018.02.010 29499228

[B4] Bohn T. Desmarchelier C. Dragsted L. O. Nielsen C. S. Stahl W. Rühl R. (2017). Host-related factors explaining interindividual variability of carotenoid bioavailability and tissue concentrations in humans. Mol. Nutr. Food Res. 61 (6), 1600685. 10.1002/mnfr.201600685 28101967 PMC5516247

[B5] Cai H. Su S. Li Y. Zeng H. Zhu Z. Guo J. (2018). Protective effects of Salvia miltiorrhiza on adenine-induced chronic renal failure by regulating the metabolic profiling and modulating the NADPH oxidase/ROS/ERK and TGF-β/Smad signaling pathways. J. Ethnopharmacol. 212, 153–165. 10.1016/j.jep.2017.09.021 29032117

[B6] Cai H. Wang J. Luo Y. Wang F. He G. Zhou G. (2021). Lindera aggregata intervents adenine-induced chronic kidney disease by mediating metabolism and TGF-β/Smad signaling pathway. Biomed. Pharmacother. 134, 111098. 10.1016/j.biopha.2020.111098 33341058

[B7] Cai X. Hua S. Deng J. Du Z. Zhang D. Liu Z. (2022). Astaxanthin activated the Nrf2/HO-1 pathway to enhance autophagy and inhibit ferroptosis, ameliorating acetaminophen-induced liver injury. ACS Appl. Mater Interfaces 14 (38), 42887–42903. 10.1021/acsami.2c10506 36094079

[B8] Chao C. T. Yeh H. Y. Tsai Y. T. Yuan T. H. Liao M. T. Huang J. W. (2020). Astaxanthin counteracts vascular calcification *in vitro* through an early Up-Regulation of SOD2 based on a transcriptomic approach. Int. J. Mol. Sci. 21 (22), 8530. 10.3390/ijms21228530 33198315 PMC7698184

[B9] Chen T. K. Knicely D. H. Grams M. E. (2019). Chronic kidney disease diagnosis and management: a review. JAMA 322 (13), 1294–1304. 10.1001/jama.2019.14745 31573641 PMC7015670

[B10] Chevalier R. L. (2016). The proximal tubule is the primary target of injury and progression of kidney disease: role of the glomerulotubular junction. Am. J. Physiol. Ren. Physiol. 311 (1), F145–F161. 10.1152/ajprenal.00164.2016 27194714 PMC4967168

[B11] Chung Y. H. Jeong S. A. Choi H. S. Ro S. Lee J. S. Park J. K. (2018). Protective effects of ginsenoside Rg2 and astaxanthin mixture against UVB-Induced DNA damage. Anim. Cells Syst. Seoul. 22 (6), 400–406. 10.1080/19768354.2018.1523806 30533262 PMC6282468

[B12] Collaborators G. B. D. D. (2024). Global age-sex-specific mortality, life expectancy, and population estimates in 204 countries and territories and 811 subnational locations, 1950-2021, and the impact of the COVID-19 pandemic: a comprehensive demographic analysis for the global burden of disease study 2021. Lancet 403 (10440), 1989–2056. 10.1016/S0140-6736(24)00476-8 38484753 PMC11126395

[B13] Cunha S. A. Borges S. Baptista-Silva S. Ribeiro T. Oliveira-Silva P. Pintado M. (2023). Astaxanthin impact on brain: health potential and market perspective. Crit. Rev. Food Sci. Nutr. 64, 1–24. 10.1080/10408398.2023.2232866 37417323

[B14] Daenen K. Andries A. Mekahli D. Van Schepdael A. Jouret F. Bammens B. (2019). Oxidative stress in chronic kidney disease. Pediatr. Nephrol. 34 (6), 975–991. 10.1007/s00467-018-4005-4 30105414

[B15] Diao W. Chen W. Cao W. Yuan H. Ji H. Wang T. (2019). Astaxanthin protects against renal fibrosis through inhibiting myofibroblast activation and promoting CD8(+) T cell recruitment. Biochim. Biophys. Acta Gen. Subj. 1863 (9), 1360–1370. 10.1016/j.bbagen.2019.05.020 31170498

[B16] Evans M. Lewis R. D. Morgan A. R. Whyte M. B. Hanif W. Bain S. C. (2022). A narrative review of chronic kidney disease in clinical practice: current challenges and future perspectives. Adv. Ther. 39 (1), 33–43. 10.1007/s12325-021-01927-z 34739697 PMC8569052

[B17] Faerber V. Kuhn K. S. Garneata L. Kalantar-Zadeh K. Kalim S. Raj D. S. (2023). The microbiome and protein carbamylation: potential targets for protein-restricted diets supplemented with ketoanalogues in predialysis chronic kidney disease. Nutrients 15 (16), 3503. 10.3390/nu15163503 37630693 PMC10459041

[B18] Foreman K. J. Marquez N. Dolgert A. Fukutaki K. Fullman N. McGaughey M. (2018). Forecasting life expectancy, years of life lost, and all-cause and cause-specific mortality for 250 causes of death: reference and alternative scenarios for 2016-40 for 195 countries and territories. Lancet 392 (10159), 2052–2090. 10.1016/S0140-6736(18)31694-5 30340847 PMC6227505

[B19] Guo H. Chen J. Yu H. Dong L. Yu R. Li Q. (2023). Activation of Nrf2/ARE pathway by anisodamine (654-2) for inhibition of cellular aging and alleviation of radiation-induced lung injury. Int. Immunopharmacol. 124 (Pt A), 110864. 10.1016/j.intimp.2023.110864 37678028

[B20] Ikee R. Sasaki N. Yasuda T. Fukazawa S. (2020). Chronic kidney disease, gut dysbiosis, and constipation: a burdensome triplet. Microorganisms 8 (12), 1862. 10.3390/microorganisms8121862 33255763 PMC7760012

[B21] Jager K. J. Kovesdy C. Langham R. Rosenberg M. Jha V. Zoccali C. (2019). A single number for advocacy and communication-worldwide more than 850 million individuals have kidney diseases. Kidney Int. 96 (5), 1048–1050. 10.1016/j.kint.2019.07.012 31582227

[B22] Jeon B. J. Yang H. M. Lyu Y. S. Pae H. O. Ju S. M. Jeon B. H. (2015). Apigenin inhibits indoxyl sulfate-induced endoplasmic reticulum stress and anti-proliferative pathways, CHOP and IL-6/p21, in human renal proximal tubular cells. Eur. Rev. Med. Pharmacol. Sci. 19 (12), 2303–2310. 26166660

[B23] Kitada M. Xu J. Ogura Y. Monno I. Koya D. (2020). Manganese superoxide dismutase dysfunction and the pathogenesis of kidney disease. Front. Physiol. 11, 755. 10.3389/fphys.2020.00755 32760286 PMC7373076

[B24] Komatsu M. Kurokawa H. Waguri S. Taguchi K. Kobayashi A. Ichimura Y. (2010). The selective autophagy substrate p62 activates the stress responsive transcription factor Nrf2 through inactivation of Keap1. Nat. Cell Biol. 12 (3), 213–223. 10.1038/ncb2021 20173742

[B25] Kumari R. Jat P. (2021). Mechanisms of cellular senescence: cell cycle arrest and senescence associated secretory phenotype. Front. Cell Dev. Biol. 9, 645593. 10.3389/fcell.2021.645593 33855023 PMC8039141

[B26] Kurosaki Y. Imoto A. Kawakami F. Ouchi M. Morita A. Yokoba M. (2022). *In vitro* study on effect of bardoxolone methyl on cisplatin-induced cellular senescence in human proximal tubular cells. Mol. Cell Biochem. 477 (3), 689–699. 10.1007/s11010-021-04295-y 34973124 PMC8857011

[B27] Lau W. L. Savoj J. Nakata M. B. Vaziri N. D. (2018). Altered microbiome in chronic kidney disease: systemic effects of gut-derived uremic toxins. Clin. Sci. (Lond) 132 (5), 509–522. 10.1042/CS20171107 29523750

[B28] Lee T. H. Chen J. J. Wu C. Y. Lin T. Y. Hung S. C. Yang H. Y. (2024). Immunosenescence, gut dysbiosis, and chronic kidney disease: interplay and implications for clinical management. Biomed. J. 47 (2), 100638. 10.1016/j.bj.2023.100638 37524304 PMC10979181

[B29] Li X. Matsumoto T. Takuwa M. Saeed Ebrahim Shaiku Ali M. Hirabashi T. Kondo H. (2020). Protective effects of astaxanthin supplementation against ultraviolet-induced photoaging in hairless mice. Biomedicines 8 (2), 18. 10.3390/biomedicines8020018 31973028 PMC7168265

[B30] Li Y. Ma K. Han Z. Chi M. Sai X. Zhu P. (2021). Immunomodulatory effects of heme Oxygenase-1 in kidney disease. Front. Med. (Lausanne) 8, 708453. 10.3389/fmed.2021.708453 34504854 PMC8421649

[B31] Liu N. Chen J. Gao D. Li W. Zheng D. (2018). Astaxanthin attenuates contrast agent-induced acute kidney injury *in vitro* and *in vivo* via the regulation of SIRT1/FOXO3a expression. Int. Urol. Nephrol. 50 (6), 1171–1180. 10.1007/s11255-018-1788-y 29368247

[B32] Loboda A. Damulewicz M. Pyza E. Jozkowicz A. Dulak J. (2016). Role of Nrf2/HO-1 system in development, oxidative stress response and diseases: an evolutionarily conserved mechanism. Cell Mol. Life Sci. 73 (17), 3221–3247. 10.1007/s00018-016-2223-0 27100828 PMC4967105

[B33] Lu Y. Liu Y. Yang C. (2017). Evaluating *in vitro* DNA damage using comet assay. J. Vis. Exp. 128. 10.3791/56450 29053680 PMC5752397

[B34] Luo L. Huang F. Zhong S. Ding R. Su J. Li X. (2022). Astaxanthin attenuates ferroptosis *via* Keap1-Nrf2/HO-1 signaling pathways in LPS-induced acute lung injury. Life Sci. 311 (Pt A), 121091. 10.1016/j.lfs.2022.121091 36252699

[B35] Naito Y. Uchiyama K. Handa O. Aoi W. (2021). Therapeutic potential of astaxanthin in diabetic kidney disease. Adv. Exp. Med. Biol. 1261, 239–248. 10.1007/978-981-15-7360-6_22 33783747

[B36] Nishida Y. Berg P. C. Shakersain B. Hecht K. Takikawa A. Tao R. (2023). Astaxanthin: past, present, and future. Mar. Drugs 21 (10), 514. 10.3390/md21100514 37888449 PMC10608541

[B37] Pan W. Kang Y. (2018). Gut microbiota and chronic kidney disease: implications for novel mechanistic insights and therapeutic strategies. Int. Urol. Nephrol. 50 (2), 289–299. 10.1007/s11255-017-1689-5 28849345

[B38] Park J. S. Choi H. I. Bae E. H. Ma S. K. Kim S. W. (2019). Paricalcitol attenuates indoxyl sulfate-induced apoptosis through the inhibition of MAPK, Akt, and NF-kB activation in HK-2 cells. Korean J. Intern Med. 34 (1), 146–155. 10.3904/kjim.2016.298 28992684 PMC6325450

[B39] Qiu X. Fu K. Zhao X. Zhang Y. Yuan Y. Zhang S. (2015). Protective effects of astaxanthin against ischemia/reperfusion induced renal injury in mice. J. Transl. Med. 13, 28. 10.1186/s12967-015-0388-1 25623758 PMC4323259

[B40] Saini R. K. Keum Y. S. Daglia M. Rengasamy K. R. (2020). Dietary carotenoids in cancer chemoprevention and chemotherapy: a review of emerging evidence. Pharmacol. Res. 157, 104830. 10.1016/j.phrs.2020.104830 32344050

[B41] Satta S. Mahmoud A. M. Wilkinson F. L. Yvonne Alexander M. White S. J. (2017). The role of Nrf2 in cardiovascular function and disease. Oxid. Med. Cell Longev. 2017, 9237263. 10.1155/2017/9237263 29104732 PMC5618775

[B42] Shen M. Chen K. Lu J. Cheng P. Xu L. Dai W. (2014). Protective effect of astaxanthin on liver fibrosis through modulation of TGF-β1 expression and autophagy. Mediat. Inflamm. 2014, 954502. 10.1155/2014/954502 24860243 PMC4016904

[B43] Si P. Zhu C. (2022). Biological and neurological activities of astaxanthin (review). Mol. Med. Rep. 26 (4), 300. 10.3892/mmr.2022.12816 35946443 PMC9435021

[B44] Sun C. Y. Lin Y. T. Huang Y. T. Cheng H. C. Chou W. C. Chang Y. T. (2025). Klotho suppresses indoxyl sulfate-mediated apoptosis in human kidney proximal tubular (HK-2) cells through modulating the AKT/Nrf2 mechanism. ACS Omega 10 (23), 24018–24029. 10.1021/acsomega.4c08038 40547630 PMC12177618

[B45] Tang H. Zhang Y. Wang Q. Zeng Z. Wang X. Li Y. (2023). Astaxanthin attenuated cigarette smoke extract-induced apoptosis via decreasing oxidative DNA damage in airway epithelium. Biomed. Pharmacother. 167, 115471. 10.1016/j.biopha.2023.115471 37699317

[B46] Vallon V. (2011). The proximal tubule in the pathophysiology of the diabetic kidney. Am. J. Physiol. Regul. Integr. Comp. Physiol. 300 (5), R1009–R1022. 10.1152/ajpregu.00809.2010 21228342 PMC3094037

[B47] Wang W. J. Cai G. Y. Chen X. M. (2017). Cellular senescence, senescence-associated secretory phenotype, and chronic kidney disease. Oncotarget 8 (38), 64520–64533. 10.18632/oncotarget.17327 28969091 PMC5610023

[B48] Xiao J. L. Liu H. Y. Sun C. C. Tang C. F. (2024). Regulation of Keap1-Nrf2 signaling in health and diseases. Mol. Biol. Rep. 51 (1), 809. 10.1007/s11033-024-09771-4 39001962

[B49] Xie X. Chen Q. Tao J. (2018). Astaxanthin promotes Nrf2/ARE signaling to inhibit HG-Induced renal fibrosis in GMCs. Mar. Drugs 16 (4), 117. 10.3390/md16040117 29621130 PMC5923404

[B50] Zhang J. Xu P. Wang Y. Wang M. Li H. Lin S. (2015). Astaxanthin prevents pulmonary fibrosis by promoting myofibroblast apoptosis dependent on Drp1-mediated mitochondrial fission. J. Cell Mol. Med. 19 (9), 2215–2231. 10.1111/jcmm.12609 26119034 PMC4568926

